# Dynamic telecytopathology of on site rapid cytology diagnoses for pancreatic carcinoma

**DOI:** 10.1186/1742-6413-3-27

**Published:** 2006-12-11

**Authors:** Burton Kim, David C Chhieng, David R Crowe, Darshana Jhala, Nirag Jhala, Thomas Winokur, Mohamad A Eloubeidi, Isam E Eltoum

**Affiliations:** 1 Scripps Green Hospital/Clinic, Department of Pathology, La Jolla, California, USA; 2 University of Alabama at Birmingham, Department of Pathology, Birmingham, Alabama, USA; 3 University of Alabama at Birmingham, Department of Gastroenterology, Birmingham, Alabama, USA

## Abstract

**Background:**

Diagnosis of pancreatic lesions can be accurately performed by endoscopic ultrasound guided fine needle aspiration (EUS-FNA) with onsite cytopathologists to assess specimen adequacy and to determine a preliminary diagnosis. Considerable time is needed to perform on-site assessments. This takes away work time of cytopathologists and prohibits them from serving remote locations. It is therefore logical to ask if real-time telecytopathology could be used to assess specimen adequacy and if telecytopathology diagnosis has the same level of agreement to the final diagnosis as that of onsite evaluation. In this study, we compare agreement between cytodiagnoses rendered using telecytopathology with onsite and final interpretations.

**Method:**

40 Diff-Quik-stained EUS-FNA were re-evaluated retrospectively (patient ages 31–62, 19:21 male:female, 15 non-malignant lesions, 25 malignant lesions as classified by final diagnosis). Each previously assessed by a cytopathologist and finally reviewed by the same or different cytopathologist. Blinded to the final diagnosis, a resident pathologist re-screened all slides for each case, selected a slide and marked the diagnostic cells most representative of the lesion. Blinded to the diagnosis, one cytopathologist assessed the marked cells through a real time remotely operated telecytopathology system (MedMicroscopy). Diagnosis and time spent were recorded. Kappa statistic was used to compare agreements between telecytopathology vs. original onsite vs. final diagnoses.

**Results:**

Time spent for prescreening ranged from 1 to 5 minutes (mean 2.6 +/- 1.3 minutes) and time spent for telecytopathology diagnosis ranged from 2–20 minutes (mean 7.5 +/- 4.5 minutes). Kappa statistics, K, was as follows: telecytopathology versus onsite diagnosis K, 95% CI = 0.65, 0.41–0.88, for telecytopathology versus final K, 95% CI = 0.61, 0.37–0.85 and for onsite diagnosis versus final K, 95% CI = 0.79, 0.61–0.98. There is no significant difference in agreement between onsite and telecytopathology diagnoses. Kappa values for telecytopathology were less than onsite evaluation when compared to the final diagnosis; however, the difference was not statistically significant.

**Conclusion:**

This retrospective study demonstrates the potential use of telecytopathology as a valid substitute for onsite evaluation of pancreatic carcinoma by EUS-FNA.

## Background

Pancreatic adenocarcinoma is a swift and deadly cancer whereupon an accurate diagnosis can be challenging, since many of the clinical and pathologic features overlap with those of non-malignant inflammatory lesions. Endoscopic ultrasound guided fine needle aspiration (EUS-FNA) has proven to be a highly sensitive and specific method to detect early malignant pancreatic lesions and to provide accurate pre-operative staging for the treatment of pancreatic adenocarcinoma [1,2]. EUS-FNA entails imaging of the pancreas from vantages of the duodenum and stomach. A cytology specimen is then aspirated through insertion of a fine needle. A cytopathologist can then examine the cells after appropriate processing of the tissue [3-8]. With smaller pancreatic lesions, the diagnostic utility of EUS-FNA exceeds that of extracorporeal ultrasound and computed tomographic aspirations [1,7].

A crucial component for the success of EUS-FNA is to have the cytopathologist examine the cellular aspirate onsite at bedside. This practice ensures accurate and consistent results for delivering a preliminary onsite diagnosis as well as ensuring that the specimen aspirated is adequate for cytological diagnosis.

When the cytopathologist participates in EUS-FNA in this manner, aconflict arises in the larger context of the cytopathology practice. For the cytopathologist, actively participating in an EUS-FNA procedure means having to interrupt one's routine work flow which results in temporarily leaving cases undiagnosed at the microscope, delaying laboratory management issues, and postponing academic administrative duties.

This practice has currently become a management issue of interest. In fact, the cost/benefit ratio of performing onsite rapid diagnoses have been scrutinized in a study by Layfield et al. where it was reported that an average fifty dollar loss per rapid onsite cytologic diagnosis performed was demonstrated when taking into account Medicare reimbursement rates (88172). Onsite rapid diagnoses adversely impacted time management for cytopathologists and subsequently lead to inadequate compensation [9].

Telepathology may reverse these findings. Telepathology has long been proposed and used to bring consultative pathology services closer by eliminating physical distances by delivering diagnoses in real-time [10]. Numerous studies have been performed validating use of telepathology by both static and dynamic systems for various surgical pathology and cytology specimens [11-17].

We propose using a dynamic telecytopathology method to eliminate traveling time, which would make better use of a cytopathologist's time and improve the cost/benefit ratio of performing rapid interpretations. Dynamic telepathology consists of a remotely operated robotic microscope that transmits digital microscopic images of glass slides from one geographic location to another in real time.

In this study we investigate the use of a dynamic telepathology system with EUS-FNA pancreatic specimens to determine if rapid diagnoses made by telepathology are just as accurate as compared to being onsite and to demonstrate an improvement of time management for the cytopathologist to further increase productivity.

## Methods

### Telepathology system

**Figure 1:**
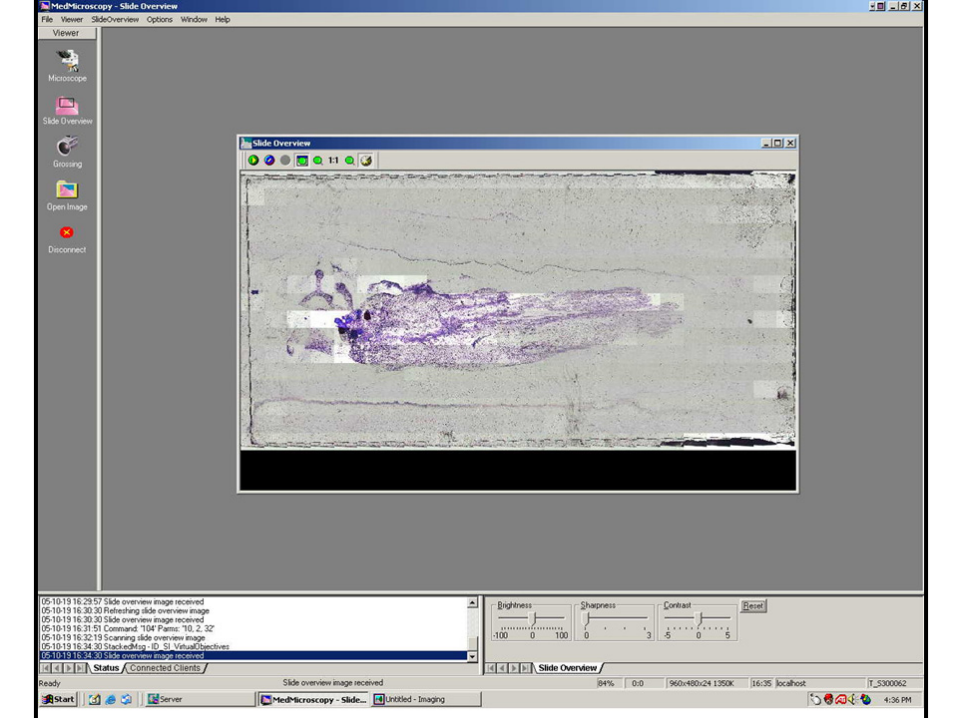
**Client Side**. Figure 1 shows the initial scan of a slide showing the whole slide. Time taken to  perform overall scan averages 2 minutes when scanning with 4x objective. Individual  squares are digital snapshots at a 24x16 grid, encompassing the entire slide.

**Figure 2:**
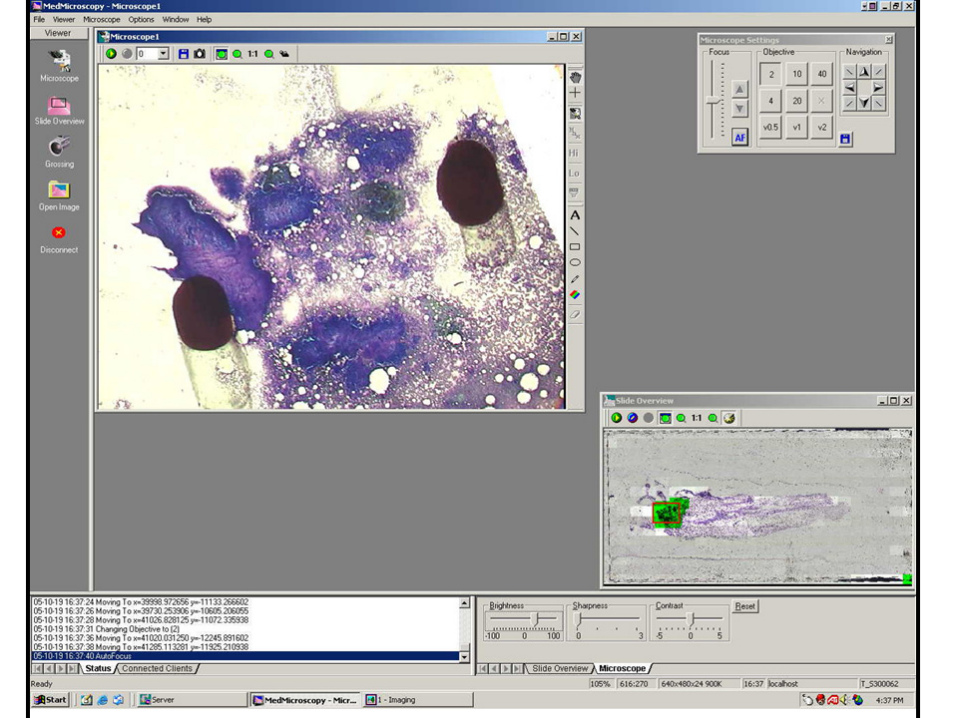
**Pre-screened Region**. Figure 2 shows further magnification of the initial whole slide scan at a pre-screened  location marked with two black ink dots.

**Figure 3:**
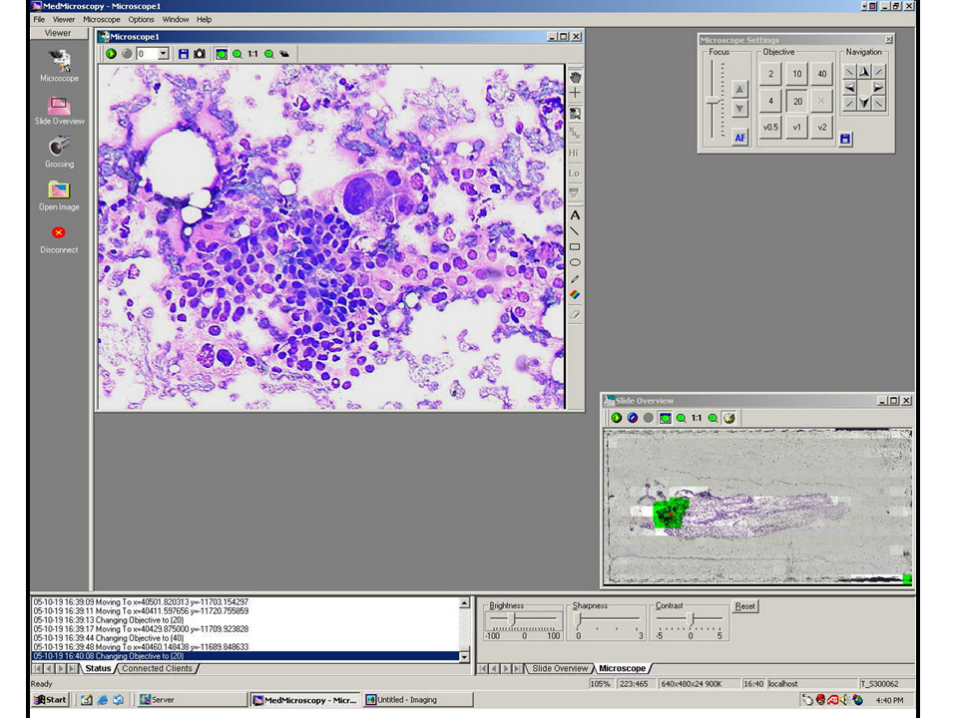
**Diagnosis**. Figure 3 shows an even higher magnification of the pre-screened region. Large  atypical cells with marked pleomorphism, abundant cytoplasm with vacuolization,  discohesiveness, and disruption of epithelial architecture are all features seen here that  illustrate the telepathology diagnosis of carcinoma.

MedMicro System (Trestle Corporation of Irvine, CA) was used for our dynamic telepathology hardware and software. This system consists of remotely operated robotics at the stage, objective, and focus levels incorporated into an Olympus BX41 microscope. This robotic system is connected to a standard PC computer (requirements: Pentium III 450 MHz, Windows 2000, 128 MB RAM, 50 MB of free Hard Drive space, 1280 × 1024 Display). At an offsite location in the cytopathologist office, remote operation was performed through a standard PC computer(requirements: Pentium 200 MMX, Windows 98/NT/2000/ME, 32 MB RAM, 10 MB of free Hard Drive space, 1024 × 768 Display) through Internet Protocol (IP). The included MedMicro Viewer software also allows full navigation of the slide, including control of objective, focus, and illumination. This system enables transfer of 24-bit images to be interpreted in a dynamic fashion in real-time (see figures 1, 2, 3).

Prior to initiating our study, an informal training session was conducted, which entailed independent familiarization with the MedMicro system that covered the basics of operation. This was regarded as minimal, since many of the functions are intuitive with point-and-click features.

### Case materials

40 pancreatic lesions diagnosed by EUS-FNA methods were retrieved. The patients' ages ranged from 31 to 62 years. There were 19 males and 21 females. Each case had been previously assessed by other cytopathologists and finally reviewed by the same or different attending cytopathologist. By previous final diagnosis, 15 cases were non-malignant (benign, inflammation, scant) and 25 were malignant (carcinoma and suspicious for carcinoma). Cover-slipped Diff-Quik-stained slides (DQ) from all cases were reviewed by a pathology resident-in-training (BK) who was blinded to the final diagnoses. Cases to be examined were created by selecting one or few (<3 slides for any given case) representative slides marked by BK to highlight areas to be then reviewed through the MedMicro system.

### Reinterpretation and specimen preparation

**Figure 4:**
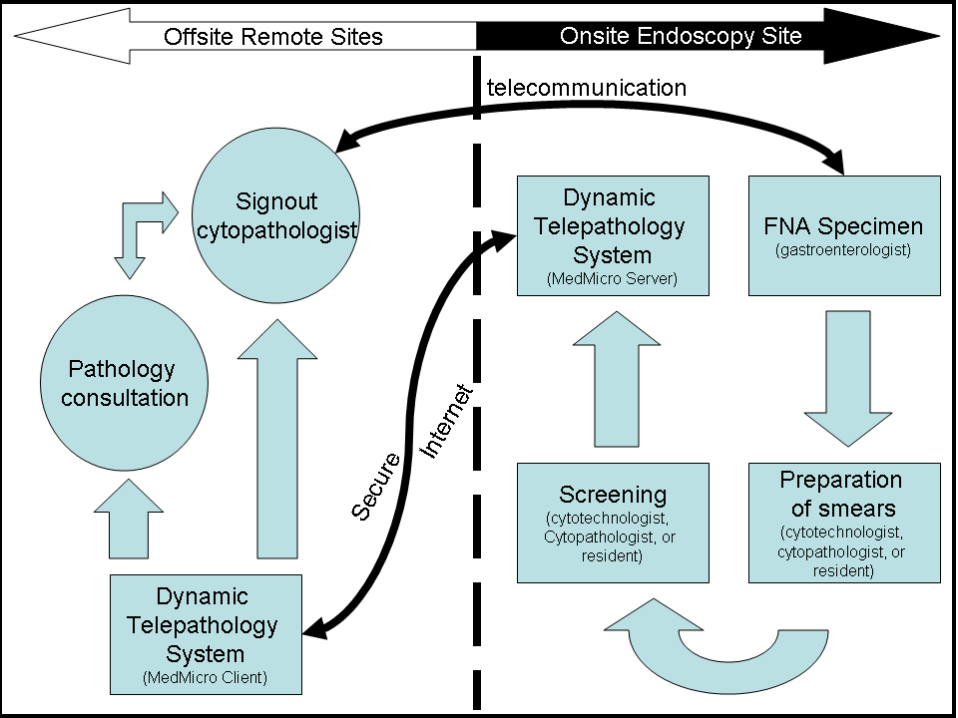
**Work Flow**. Figure 4 shows the telepathology workflow which starts at the FNA aspiration by the  gastroenterologist. The specimen is then prepared and corresponding slides are prescreened.  Real-time interpretation of the slides can then be performed from single or  multiple end user cytopathologists who may diagnosis as solo or as a consensus  group. Results can then be given by telephone or intercom to the gastroenterologist  onsite. This work diagram can be implemented in most any geographic situation.

The selected pre-screened DQ slides were randomized to be presented in an unknown fashion through the MedMicro system for an offsite attending cytopathologist (IAE). The selected slide(s) was placed onto the stage of the robotic microscope by BK and an initial whole slide scan was performed, encompassing approximately two minutes for each case. Once the slide has been initially pre-scanned, the cytopathologist then re-evaluated the cases by remotely controlling and reviewing the prescreened, randomized cases. The slide could be magnified, focused, and moved at the discretion of the offsite cytopathologist. Diagnoses were then rendered for each case using categories of negative for malignancy, positive for malignancy, and suspicious for malignancy. For this study the cytopathologist was also blinded to the original demographics and diagnoses of these cases (see figure 4).

### Statistics

Data was collected and collated using an Excel spreadsheet. Kappa statistic was used to compare agreements between telepathology versus original onsite and versus final diagnoses. Times were recorded with standard calculations of averages and standard deviation.

## Results

Using the MedMicro system, the attending cytopathologist diagnosed 18 of the 40 cases negative for carcinoma, 16 positive for carcinoma, and 6 suspicious for carcinoma. The previous onsite preliminary diagnoses had been 18 for carcinoma, 16 for benign/scant/inflammation, and 6 for suspicious. Final diagnoses of the same cases showed 23 for carcinoma, 15 for benign/inflammation, and 2 for suspicious. We combined these findings into two categories of benign and malignant while defining carcinoma and suspicious for carcinoma as both malignant. The remainder was defined as benign. With these two categories established, Kappa statistic calculations were performed. Kappa for telepathology versus onsite rapid diagnosis was 0.65, 0.41–0.88 (K, 95% CI). Kappa for telepathology versus final diagnosis was 0.61, 0.37–0.85 (K, 95% CI). Kappa for onsite rapid diagnosis versus final diagnosis was 0.79, 0.61–0.98 (K, 95% CI).

Time spent by the resident-in-training for prescreening and selecting example slides from each of the 40 cases ranged from 1 to 4 minutes (mean 2.6 ± 1.3 minutes). Overall, less time was spent on those cases with fewer original slides to preview and more time was needed to prescreen those cases with more original slides. Time spent to make a telepathology diagnosis ranged from 2–20 minutes (mean 7.5 ± 4.5 minutes). This time does not include the initial whole slide pre-scan, which would add two minutes to each case. The longer telepathology diagnostic times were noted in the beginning at the start of this study, and as our study progressed, the time needed to reach a diagnosis decreased.

Overall, the quality of images was considered excellent as surveyed by the remote cytopathologist. The effort to operate the robotic microscope from an offsite location was also noted to be non-problematic. Occasional fine-tuning was needed at the microscope site for initial setup of analog light intensity and condenser position. Once the microscope was configured to optimally view slides, no further adjustments were performed.

## Discussion

EUS-FNA is superior to other methods in overall cost/benefit aspects for diagnosing pancreatic carcinomas [18,19]. Together with both the endoscopist and cytopathologist onsite, EUS-FNA provides high cellular yield (86%–98%), high sensitivity (77%–95%) and high specificity (96%–100%). Overall accuracy (79%–97%) has been shown for the diagnosis of malignant neoplasia. Also, previous studies have shown a Kappa value of 0.88 when comparing onsite EUS-FNA rapid diagnoses to offsite final diagnoses for all pancreatic lesions. With regards to diagnoses of carcinoma, a kappa value of 1.0 was also reported [4,7,18-21].

A major reason why EUS-FNA is so successful is because an onsite cytopathologist is present to process and interpret. It has been shown that specimen yield is better whenever a cytopathologist is onsite versus not being present [2,22], but postponing other duties to be onsite at a EUS-FNA comes at a cost to the cytopathologist. Our study suggests that telepathology is an appropriate surrogate for being onsite, while saving time.

In support of the previous studies noting the high accuracy of EUS-FNA, we found similar results in our study through use of our dynamic telepathology system. We also calculated a kappa value of 0.79 when comparing onsite EUS-FNA rapid diagnoses to offsite final diagnoses for all pancreatic lesions, benign and malignant. Kappa value was also 1.0 with carcinoma diagnoses.

Since statistically our control group agreed with previous study results, we deemed our comparison data (onsite rapid vs. final) reliable to use when investigating the effectiveness of telepathology. The Kappa value of telepathology diagnoses with that of the final diagnoses was 0.61 [sensitivity = 0.86 (0.76–1.00), specificity = 0.67 (0.45–0.88), PPV = 0.76 (0.59–0.93), NPV = 0.80 (0.60 – 1.00). When comparing onsite rapid (Kappa-rapid = 0.79) and offsite telepathology (Kappa-telepathology = 0.61) as referenced to the final diagnoses, no significant difference in agreement was noted.

Indeed Kappa values for telecytopathology were less than onsite evaluation when compared to the final diagnosis, even though this difference was not statistically significant. There are several possible reasons for this. Final diagnoses had the advantages of Papanicolaou stain slides, hematoxylin/eosin stained cell block preparations, and other ancillary tests such as flow cytometry to aid in a more comprehensive diagnosis. Originally, all the DQ slides in these cases were created onsite and the onsite cytopathologist had the advantage of looking through all the DQ slides of a given case to aid in the onsite rapid diagnosis.

For our telepathology arm of this study the cases were pre-screened and only one to three slides were selected from each case. Many cases were limited to one selected DQ slide with the assumption that the single slide would be truly representative for the entire case. Therefore, prescreening and selecting the single most pertinent slide weighed heavily upon the skill and experience of the prescreener, BK, whose job was to mark any atypical cells. In this study, it may be noteworthy to mention that BK had no prior formal cytology training at the time of pre-screening.

Familiarity with the telepathology system and being accustomed to viewing diagnostic images on a computer monitor may also have played a role in a decreased correlation. Many of the false-positives and false-negative cases rendered through the telepathology method were made early within the 40 cases seen. When the cytopathologist became more accustomed to the MedMicro system, we noted fewer discordant diagnoses as well as shorter diagnosis times.

Other studies with telepathology have been performed with success, detailing the utility of telepathology with cytology in other organs, such as breast, gastrointestinal, and gynecological [23-28]. Most of these studies involved static telepathology methods where upon a diagnosis is made on one or few images. Marchevsky et al has explored with encouraging results the use of static telepathology techniques on EUS-FNA pancreatic carcinoma specimens [29]. The drawback of a static telepathology method is the image sampling. Is the snapshot truly representative for the whole slide? For cytology, cells are often arranged in a three dimensional manner and the examination of multiple focal planes are crucial to an accurate diagnosis. Also, cells are spread throughout the slide, and scanning an entire slide is necessary to ensure that all relevant cells are examined.

A common bottleneck for both static and dynamic telepathology is data processing and transmission speeds. Most institutions operate on a T1 speed line (1.544 Mbps) Microscopic digital images of surgical pathology and cytology are complex and indeed generate large file sizes, which lead to transmission times too long to be deemed useful.

Four other main issues come into play regarding use of a dynamic telepathology system as a substitute for onsite rapid diagnosis of EUS-FNA cases. The first issue regards accuracy. When comparing a cytopathologist using telepathology coupled with an onsite prescreener versus a regular onsite cytopathologist, our study showed statistically non-significant decrease in observed Kappa values between telepathology and onsite rapid diagnoses. Even though Kappa for telepathology versus final diagnosis was less than that of onsite rapid values, it still demonstrates how telepathology can be an appropriate substitution. In fact, combined with improved onsite pre-screening and competency in telepathology utilization, accuracy should only increase to better match those of onsite evaluations. However, additional prospective studies on this proposal must be performed.

The second issue regards the actual benefit of preventing time from being spent away from routine office duties. For the telepathology method when summing the pre-screen time, scan time, and diagnosis time, an average total of 12 minutes is needed to perform a telepathology rapid diagnosis. An average of 7.5 minutes of this total is actually spent by the cytopathologist in rendering a rapid diagnosis through telepathology. This is much shorter than the time spent for the cytopathologist who must travel to the site and wait during the dead time between aspirations, which can total to average over 30 minutes [30]. Also, with the time saved by the telepathology cytopathologist, routine office work can continue with improved time management.

The third issue regards to loss of personal contact and communication with the clinician that may damage patient care. Our study suggests that it is not essential for a cytopathologist to be physically present at bedside for rapid diagnoses. However, this does not mean all or any interaction with the endoscopist is sacrificed. Indeed, a crucial component to our telepathology method is communication. Telepathology is highly interactive where multiple simultaneous users can access it to share their thoughts on each case. The ability for mass communication/teaching beyond any distance is the major advantage gained for the sacrifice of the physical interaction between the pathologist and clinician. In fact, telepathology advances communication so that pathologists can reach out to more clinicians, thereby improving patient care as a whole.

The fourth issue regards startup capital costs. Even though the cytopathologist is not physically present onsite, our dynamic telepathology method still requires a member with sufficient experience to pre-screen and prepare slides. Costs of this member with expertise would certainly need to be included when assessing the overall cost/benefit ratio of this telepathology method. Current thought is that onsite rapid diagnosis of EUS-FNA cases entails a loss in revenue, resulting in an unprofitable use of a cytopathologist's time, since average time taken during one onsite event may average to over an hour [30]. Also, startup costs of additional equipment and time taken to learn a new method are both issues to take into account to evaluate the overall cost effectiveness of telepathology. However, once a system is in place and efficient competency is achieved, a telepathology practice can be more cost effective and lead to more opportunities.

## Conclusion

EUS-FNA of pancreatic malignancies has been proven to be accurate and cost effective, especially when an onsite cytopathologist is present. Our study demonstrates that telecytopathology has statistically equal accuracy to onsite rapid examinations. It also emphasizes the additional advantages of time and cost savings of using telecytopathology with EUS-FNA. Telecytopathology is an appropriate substitute for the onsite evaluation of pancreatic EUS-FNA and aids cytopathologists to use time more efficiently.

## Competing interests

The author(s) declare that they have no competing interests.

## Authors' contributions

BK pre-screened all cases and managed the server component of the robotic microscope. IAE examined each case on the client side and rendered new telepathology diagnoses. For the original cases MAE aspirated the original specimens. TW oriented us to the telepathology system. The remainder of the authors contributed through diagnosing the cases prior with both rapid and final diagnoses. All authors read and approved the final manuscript.
